# Clinical Assessment of Gingival Sulcus Depth and Biological Width Among a Population of Young Moroccan Adults

**DOI:** 10.7759/cureus.111807

**Published:** 2026-06-30

**Authors:** Hanane El Bekkali, Imane El Bassity, Sara Benfaida, Oumaima Labzar, Khadija Amine, Mouna Hamza, Anas Bennani

**Affiliations:** 1 Department of Fixed Prosthodontics, Faculty of Dental Medicine of Casablanca, Hassan II University of Casablanca, Casablanca, MAR; 2 Department of General Dentistry, Faculty of Dental Medicine of Casablanca, Hassan II University of Casablanca, Casablanca, MAR; 3 Department of Periodontology, Faculty of Dental Medicine of Casablanca, Hassan II University of Casablanca, Casablanca, MAR; 4 Department of Pediatric Dentistry, Faculty of Dental Medicine, Mohammed VI University of Health Sciences, Casablanca, MAR

**Keywords:** biological width, diagnostic imaging, gingival sulcus depth, periodontal index, periodontics

## Abstract

Objective

The gingival sulcus and biological width are key anatomical structures involved in maintaining periodontal health and guiding prosthodontic treatment planning. Although their clinical importance has been well established, data describing these parameters in Moroccan populations remain limited. This study aimed to evaluate the mean gingival sulcus depth and biological width among young Moroccan adults and to provide baseline descriptive data for future periodontal assessment and treatment planning.

Methods

A descriptive cross-sectional study was conducted involving 133 dental students (72 males and 61 females), aged 21 to 23 years, at the Dental Consultation and Treatment Center (CCTD) of Ibn Rochd University Hospital, affiliated with the Faculty of Dental Medicine of Casablanca (FMDC), Casablanca, Morocco. Each participant underwent standardized clinical and radiographic examinations of one anterior and one posterior tooth. Sulcus depth was measured using a calibrated periodontal probe, while biological width was assessed using periapical radiographs. A single calibrated examiner performed all measurements following a standardized protocol. Data were analyzed using IBM SPSS Statistics for Windows, Version 20.0 (IBM Corp., Armonk, NY, USA), and mean values were compared using Student’s t-test.

Results

The mean sulcus depth was 1.11 mm for anterior teeth and 2.17 mm for posterior teeth, with a statistically significant difference (p < 0.0001). The mean biological width was 2.05 mm, consistent with classical reference values reported in the literature. No statistically significant sex-related differences were observed for either parameter. Sulcus depth was significantly greater in posterior teeth than in anterior teeth (p < 0.0001), and biological width differed significantly between anterior and posterior sites (p = 0.003).

Conclusions

The findings are consistent with previously reported values in the literature and confirm the relative stability of biological width in healthy periodontal tissues. These findings provide descriptive anatomical data that may contribute to biologically informed prosthodontic treatment planning. Further studies incorporating additional periodontal parameters and more diverse populations are recommended to improve external validity.

## Introduction

The long-term success of fixed prosthodontic treatments relies on the preservation of periodontal health through a harmonious and biologically respectful integration of dental, periodontal, and prosthetic components [1]. Central to this equilibrium are two anatomical structures of paramount importance: the gingival sulcus and the biological width [2-4].

The gingival sulcus is the anatomical space between the tooth surface and the sulcular epithelium, coronally bounded by the free gingival margin [5]. In clinically healthy individuals, its depth typically ranges from 0.5 to 3.0 mm, although this value may vary depending on individual factors [6]. An increased sulcus depth may indicate early inflammatory changes, while a marked reduction is often associated with gingival recession [7,8].

Complementing this, the biological width refers to the supracrestal soft tissue attachment located between the alveolar bone crest and the base of the sulcus. It comprises two histological components: the junctional epithelium (approximately 0.97 mm) and the connective tissue attachment (approximately 1.07 mm), for a combined average height of about 2.04 mm [3,9]. This dimension acts as a protective barrier against microbial and mechanical insults. Violation of the biological width, particularly through subgingival placement of restoration margins during fixed prosthetic procedures, has been associated with a variety of complications, including chronic inflammation, marginal bone loss, and gingival instability [2-12].

Therefore, precise assessment of both sulcus depth and biological width is essential. These parameters are not only fundamental to periodontal evaluation but also serve as critical determinants in the design, placement, and long-term prognosis of prosthetic restorations [13-15].

Although the clinical importance of preserving these anatomical dimensions has been well established by studies such as those conducted by Gargiulo et al. [3], Padbury et al. [14], Vacek et al. [15], and Kois [16], the existing data are largely derived from non-Moroccan or heterogeneous populations. There is a notable lack of descriptive data regarding gingival sulcus depth and biological width among Moroccan young adults, especially within academic environments. This gap highlights the need for context-specific investigations to inform evidence-based clinical decision-making and enhance the relevance of periodontal assessment protocols.

This study, therefore, aimed to evaluate the mean gingival sulcus depth and mean biological width among dental students at the Faculty of Dental Medicine of Casablanca (FMDC), Casablanca, Morocco, and to compare these measurements according to tooth location and sex. By providing baseline descriptive periodontal data for this study population, the study aims to support biologically informed prosthetic planning and contribute to evidence-based clinical education tailored to the local context.

## Materials and methods

The present descriptive cross-sectional study was conducted between February 12, 2024, and January 26, 2025, in the Department of Fixed Prosthodontics at the Center for Dental Consultations and Treatments (CCTD) of Ibn Rochd University Hospital, affiliated with the FMDC.

This study was approved by the Ethics Committee of Hassan II University, FDMC (approval number: FMDC-pr2c/54-2025). The study was conducted in accordance with the ethical principles of the Declaration of Helsinki. Written informed consent was obtained from all participants prior to data collection. All collected data were anonymized to ensure participant confidentiality, and subjects were informed of their right to withdraw from the study at any time without consequence.

Study participants

The study population consisted of dental students. A complete list of students and their clinical rotation schedules across the various departments of the CCTD was obtained from the faculty administration to facilitate the planning and coordination of data collection procedures. Participants were screened according to predefined inclusion and exclusion criteria, which are summarized in Table 1.

**Table 1:** Inclusion and exclusion criteria for study participant selection

Inclusion Criteria	Exclusion Criteria
Age between 21 and 23 years	Age outside the study age range
Clinically healthy periodontium or previously treated stable periodontium confirmed by clinical re-evaluation	Significant systemic medical conditions
Good oral hygiene assessed using standardized clinical indices	Pregnancy
No history of mucogingival surgery	History of mucogingival surgery
No history of orthodontic treatment	History of orthodontic treatment
No use of medications associated with gingival overgrowth (e.g., phenytoin, cyclosporine, calcium channel blockers)	Use of medications associated with gingival overgrowth (e.g., phenytoin, cyclosporine, calcium channel blockers)
Teeth free of cervical restorations, active carious lesions, and fixed prosthetic restorations	Presence of cervical restorations, active carious lesions, or fixed prosthetic restorations on the teeth selected for examination

Measurement of the gingival sulcus depth

For each participant, gingival sulcus depth was assessed at six sites on two natural teeth: three buccal sites (mesiobuccal, mid-buccal, distobuccal) and three corresponding palatal or lingual sites. The teeth selected for evaluation included one anterior tooth, either a central or lateral incisor (maxillary or mandibular), and one posterior tooth, a first molar (maxillary or mandibular). This selection was intended to provide representative data from both anterior and posterior regions of the oral cavity, thereby enabling relevant clinical and radiographic comparisons.

A calibrated University of North Carolina (UNC)-15 periodontal probe was used to perform the measurements. All probing depths were recorded in millimeters and documented using standardized clinical recording forms. Probing depth was used as a clinical surrogate of sulcus depth.

Measurement of biological width

Biological width was evaluated using periapical radiographs obtained with the parallel technique, employing a positioning device to ensure reproducibility and minimize distortion. The measurement was defined as the vertical distance between the cementoenamel junction (CEJ) and the most coronal point of the alveolar bone crest. This radiographic measurement represents a CEJ-to-alveolar crest distance used as a surrogate for biological width rather than a direct histological measurement of supracrestal tissue attachment.

Data recording and statistical analysis

All clinical and radiographic measurements were recorded on standardized clinical data sheets. A single examiner specializing in periodontology performed all measurements following a standardized protocol to ensure consistency and reproducibility. However, intra-examiner reliability was not formally assessed, which may represent a limitation of the study.

Data were analyzed using IBM SPSS Statistics for Windows, Version 20.0 (IBM Corp., Armonk, NY, USA). Quantitative variables were expressed as means and standard deviations, while qualitative variables were reported as frequencies and percentages. Comparative analyses were conducted between anterior and posterior teeth, as well as between buccal and palatal/lingual surfaces. Additional comparisons were performed according to participant sex. Student’s t-test was employed to compare mean values, with statistical significance set at α = 0.05. For each tooth, mean values were calculated from six measurement sites.

## Results

A total of 133 dental students participated in the study, representing 44.5% of the eligible population (n = 299). The mean age of the participants was 22.2 ± 0.6 years (range: 21-23 years). The sample included 61 females (45.9%) and 72 males (54.1%).

The clinical measurements of sulcus depth and biological width were recorded on both anterior and posterior teeth. Posterior teeth exhibited significantly greater sulcus depths than anterior teeth (p < 0.0001), highlighting anatomical differences between these regions (Table 2). The mean biological width measured in the study population was 2.05 mm, with similar values at mesial and distal sites, indicating relatively low intra-individual variability within this standardized sample (Table 3).

**Table 2:** Mean sulcus depth for anterior and posterior teeth.

Surface	Mean (mm)	Standard Deviation (mm)	Minimum (mm)	Maximum (mm)
Anterior teeth				
Mesio-buccal	1.1917	0.54952	0.5	3
Mid-buccal	0.8496	0.40351	0.5	3
Disto-buccal	1.3571	0.70596	0.5	4
Mesio-palatal	1.1617	0.53848	0.5	3
Mid-palatal	0.9135	0.44599	0.5	2
Disto-palatal	1.1917	0.51390	0.5	3
Posterior teeth				
Mesio-buccal	2.1955	0.65665	1.0	4.0
Mid-buccal	1.6429	0.69786	0.5	3.5
Disto-buccal	2.2406	0.72457	1.0	5.0
Mesio-palatal	2.3647	0.74653	1.0	4.0
Mid-palatal	1.8947	0.68549	0,5	3.0
Disto-palatal	2.6617	0.66443	1.0	5.0

**Table 3:** Mean biological width for anterior and posterior teeth.

Parameter	Anterior Tooth (mm)	Posterior Tooth (mm)	Overall (mm)
Mean-Mesial	2.0196	2.0831	2.0477
Standard deviation-Mesial	0.19246	0.09405	0.15929
Minimum-Mésial	1.0	2.0	1.0
Maximum-Mesial	2.5	2.3	2.5
Mean-Distal	2.0424	2.0754	2.0571
Standard deviation- Distal	0.14958	0.09067	0.1275
Minimum- Distal	1.8	2.0	1.8
Maximum- Distal	2.4	2.3	2.4

When comparing sulcus depth by sex, no statistically significant differences were observed across all measurement sites (Table 4). In terms of biological width, no statistically significant differences were observed between males and females, with comparable mean values at both mesial and distal sites. However, a statistically significant difference was observed between anterior and posterior teeth (p =0.003) (Table 4).

**Table 4:** Comparison of mean sulcus depth and biological width by sex (n = 133).

Anatomical Surface/Parameter	Male (N=72; Mean±SD; mm)	Female (N=61; Mean ± SD; mm)	p-value
Mesio-buccal surface (anterior)	1.22 ± 0.54	1.18 ± 0.55	0.6742
Mid-buccal surface (anterior)	0.85 ± 0.41	0.85 ± 0.41	1.0
Disto-buccal surface (anterior)	1.38 ± 0.70	1.33 ± 0.71	0.6845
Mesio-palatal surface (anterior)	1.20 ± 0.56	1.13 ± 0.52	0.4566
Mid-palatal surface (anterior)	0.89 ± 0.46	0.93 ± 0.48	0.6263
Disto-palatal surface (anterior)	1.17 ± 0.52	1.21 ± 0.50	0.6525
Mesio-buccal surface (posterior)	2.25 ± 0.66	2.14 ± 0.65	0.336
Mid-buccal surface (posterior)	1.65 ± 0.68	1.63 ± 0.70	0.8681
Disto-buccal surface (posterior)	2.28 ± 0.74	2.20 ± 0.71	0.5265
Mesio-lingual surface (posterior)	2.39 ± 0.76	2.33 ± 0.73	0.6438
Mid-lingual surface (posterior)	1.92 ± 0.68	1.86 ± 0.71	0.6214
Disto-lingual surface (posterior)	2.70 ± 0.66	2.63 ± 0.67	0.5466
Mesial biological width	2.06 ± 0.14	2.03 ± 0.17	0.2743
Distal biological width	2.07 ± 0.12	2.05 ± 0.13	0.3616

## Discussion

The clinical significance of gingival sulcus depth and biological width is well established in both periodontology and fixed prosthodontics, particularly regarding the preservation of the dento-gingival interface during restorative or surgical procedures. Maintaining this balance is essential for tissue health and long-term treatment success. Respecting the biological width has been consistently associated with the long-term success of restorative treatments, as violations of the supracrestal attachment may lead to chronic inflammation, marginal bone loss, and aesthetic compromise. These observations are consistent with classical prosthodontic principles, suggesting that adequate space between the restoration margin and the alveolar bone crest is important to preserve periodontal health and tissue stability [2].

In the present study, gingival sulcus depth was found to be significantly greater in posterior teeth (2.17 mm) compared to anterior teeth (1.11 mm) (p < 0.0001). This finding is consistent with previous reports by Rijal et al. [10], Al-Rasheed et al. [17], and Shirmohammadi et al. [18], likely due to more complex root morphology and thicker tissue in posterior regions. This finding supports clinical guidelines advising site-specific restoration margin placement to avoid biological width violation [14].

No statistically significant differences in sulcus depth were observed between male and female participants. This supports the concept that sulcus depth in young, periodontally healthy individuals is relatively independent of sex, in agreement with findings reported by Rajesh et al. [9] and Rijal et al. [10].

Regarding biological width, originally defined by Gargiulo et al. as the combined height of the junctional epithelium and connective tissue attachment, averaging 2.04 mm [3], our mean value of 2.05 mm aligns closely with previously reported values in the literature reported by Vacek et al. [15] and Al-Rasheed et al. [17]. However, this value corresponds to a radiographic CEJ-to-alveolar crest distance used as a surrogate for biological width and should be interpreted as a radiographic approximation rather than a direct histological measurement, which may partly explain differences with classical anatomical definitions.

No statistically significant differences were found in biological width measurements between males and females in our sample. Mean values at the mesial and distal sites were very similar across sexes, indicating morphological consistency of this parameter. These findings align with those of Vacek et al. [15], Al-Rasheed et al. [17], Zweers et al. [19], and Kolte et al. [20]. This supports the concept that biological width is a relatively constant anatomical entity, with variations more related to pathological or iatrogenic factors than individual characteristics like sex.

However, a statistically significant difference in biological width was observed between anterior and posterior sites. This variation is well documented by Vacek et al., with posterior teeth generally exhibiting slightly greater biological width due to more complex root anatomy and thicker local tissue [15]. Respecting this site-specific architecture is crucial for peri-prosthetic tissue stability, as emphasized by Ingber et al. [11] and Lang and Lindhe [21].

To ensure methodological validity, the present study was conducted in accordance with a rigorous methodological framework, grounded in established clinical and scientific standards. The present study, conducted on a sample of 133 dental students, is characterized by a sample size allowing statistical analysis of the studied parameters. This aligns with the methodological recommendations of Vacek et al., who underscored the necessity of an adequately powered sample for the reliable assessment of periodontal parameters, particularly sulcular depth and biological width dimensions [15].

The selection of a homogeneous population of periodontally healthy young adults represents a methodological strength, as it minimizes the impact of confounding variables such as age, systemic health, or previous periodontal conditions. This approach is supported by Zweers et al. and Kolte et al., who emphasized the importance of controlling for such factors in periodontal research [19,20]. However, this same homogeneity also constitutes a limitation, as it restricts the external validity of the findings. The results may not be generalizable to older individuals or patients with pre-existing periodontal conditions.

The measurement protocol followed in this study adheres closely to established methodological standards and incorporates validated reference instruments. Sulcular depth was measured using a calibrated UNC-15 periodontal probe, recognized for its precision and reproducibility [22]. Biological width was assessed through conventional periapical radiographs obtained via the parallel technique, a method shown to reliably measure the vertical distance between the CEJ and the alveolar crest [3,17]. However, intra-examiner reliability was not formally assessed, which may limit the reproducibility of the measurements.

Although emerging digital technologies offer enhanced accuracy and reliability, as noted by Monaco et al. [23], the use of conventional techniques in this study remains fully justified. When applied under standardized and controlled conditions, these traditional methods provide satisfactory reproducibility and diagnostic validity.

Comparisons between anterior and posterior teeth were emphasized to reflect clinically relevant anatomical and functional differences, supporting recommendations by Padbury et al. [14] and confirmed by Rijal et al. [10].

To ensure methodological feasibility and minimize variability due to tooth-specific anatomical differences, the analysis was restricted to a single anterior and a single posterior tooth per participant. While this strategy is methodologically sound, it nonetheless represents a limitation. Additionally, the study did not account for potentially influential variables such as gingival thickness or the precise inflammatory status of the periodontal tissues. These aspects warrant further investigation in future studies involving more heterogeneous populations, including individuals with active periodontal disease or those who have undergone orthodontic or prosthetic treatment.

The absence of additional periodontal parameters such as gingival thickness, clinical attachment level, bleeding on probing, and plaque index also represents a limitation of the present study.

Despite limitations, this study provides novel quantitative data on the gingival sulcus and biological width dimensions in healthy young adults. By focusing on a Moroccan student population, it enriches the scientific literature with contextualized data previously underrepresented.

The results confirm the relative stability of biological width dimensions while highlighting significant differences based on tooth location. These findings are consistent with the anatomical rationale underlying the classical 3 mm restorative guideline reported in the literature, although this study did not directly evaluate restorative margins or test this clinical threshold. These results should be interpreted as radiographic surrogate measurements rather than direct biological width values. Future research should focus on validating these findings in more diverse populations, including patients with periodontal disease or those undergoing orthodontic or prosthetic treatments. The integration of advanced digital tools for assessing biological width could also represent a valuable development in both diagnosis and therapy.

## Conclusions

This study demonstrated that the gingival sulcus depth was significantly greater in posterior teeth than in anterior teeth, while no significant differences were observed between male and female participants. The findings should be interpreted in light of the radiographic assessment method used, which provides a surrogate measurement of the CEJ-to-alveolar crest distance rather than a direct histological evaluation of biological width.

The findings should, therefore, be considered descriptive baseline data for this specific population rather than generalizable clinical reference values. These findings contribute to the understanding of periodontal anatomy and may support biologically informed prosthodontic treatment planning. Further studies involving larger and more diverse populations are needed to validate and expand these observations.
